# Fungal and Bacterial Diversity Isolated from *Aquilaria malaccensis* Tree and Soil, Induces Agarospirol Formation within 3 Months after Artificial Infection

**DOI:** 10.3389/fmicb.2017.01286

**Published:** 2017-07-11

**Authors:** Hemraj Chhipa, Nutan Kaushik

**Affiliations:** Plant Biotechnology, The Energy and Resources Institute New Delhi, India

**Keywords:** endophytes, diversity, artificial infection, *Pantoea dispera*, agarwood, agarospirol, GC-MS

## Abstract

*Aquilaria malaccensis* Lam, commonly known as Agarwood, is a highly valuable species used in production of agar oil from its infected wood, which is utilized in pharmaceutical and perfumery industry. Agar oil formation in agarwood takes years through the natural process which is induced by natural or artificial injury or microbial infection. The role of soil fungi and bacteria in artificial induction is still an unexplored area. In the present study, we isolated the fungal and bacterial community residing inside the stem of *A. malaccensis* tree and circumventing soil, samples collected from 21 different sites of the north-eastern state Assam of India and explored their potential in induction of Agarospirol (2-(6,10-Dimethylspiro[4,5]dec-6-en-2-yl)-2-propanol) production by artificially infecting the trees with these microorganisms. A total 340 fungi and 131 bacteria were isolated from 50 stem samples, and 188 fungi and 148 bacteria were isolated from 50 soil samples. Highest Shannon (*H*′ = 2.43) and Fisher (α = 5.57) diversity index was observed in the stem isolates. The dominant fungal genus was *Trichoderma* in stem with *Pi* value of 0.18; while in soil, *Aspergillus* showed dominance with *Pi* value 0.73. In bacteria, *Bacillus* genera showed dominance in both stem and soil samples with *Pi* = 0.62 and 0.51, respectively. Forty fungal and bacterial isolates were used to assess their potential to induce formation of agarwood in *A. malaccensis* by artificial infection method. Gas chromatography mass spectroscopy (GC-MS) analysis confirmed development of Agarwood by the presence of Agarospirol compound in samples collected after 3 months of the artificial infection. Only 31% of bacterial and 23% of fungal isolates showed their ability in production of Agarospirol by artificial infection method. Bacteria *Pantoea dispersa* and fungi *Penicillium polonicum* showed the highest production in comparison to other isolates.

## Introduction

*Aquilaria malaccensis* is an oleoresin-producing tree which occurs in Malaysia, Myanmar, Sumatra, Borneo Island, Philippines, Bangladesh, Vietnam, China, and India (Gibson, 1977; Hai et al., 1999). The oleoresin produced by the tree is a highly valuable component of incense, perfumes, drugs, stimulants, cardiac tonics, and carminatives (Mengling et al., 2005). Further, it is used as an active ingredient in traditional medicines of China and has also been included in different pharmaceutical products of coughs, acroparalysis, asthma, and anti-histamine (Kim et al., 1997; Bhuiyan and Bhuiyan, 2008). The dark wood of *Aquilaria* tree having oleoresin is known as agarwood, which emits a pleasant smell on burning and is widely used as incense in Islamic, Buddhist, and Hindu ceremonies. The essential oil extracted from agarwood also has antimicrobial properties (Chen et al., 2011). Commercial exploitation and uncontrolled cutting is leading to a decline in number of these trees. Eight species of *Aquilaria* are listed in Appendix II of the Convention on International Trade in Endangered Species (CITES) of wild fauna and flora (CITES, 1994) and are categorized as vulnerable by the International Union for the Conservation of Nature (IUCN, 2009).

Agarwood is the oleoresin containing part of *Aquilaria* tree which is formed after natural and artificial injury and tree produced oleoresin to prevent or recover the injury in response to plant defense mechanism (Zhang et al., 2012). Naturally the wound is produced by microbial invasion, gnawing of insects, lightning strikes and heavy winds (Xu et al., 2013; Zhang et al., 2014). As a defense response to these outbreaks, normal heartwood converts into dark agarwood. Firstly, Bhattacharyya in 1952 reported role of endophytic fungi in inducing agarwood production in the tree trunk. Later many scientists isolated several endophytes from the agarwood tree (Chhipa et al., 2017). Mohamed et al. (2010) showed the presence of *Curvularia, Cunninghamella, Trichoderma*, and *Fusarium* species in the agarwood fungal community in Malaysia. Similarly, Tian et al. (2013) reported the presence of *Phomopsis, Botryosphaeria, Cylindrocladium*, and *Colletotrichum gloeosporioides* species in wounded *Aquilaria* tree; and the presence of *Alternaria, Mycosphaerella, Phoma, Ramichloridium*, and *Sagenomella* species in the non-resinous tree internally. Bhore et al. (2013) demonstrated the presence of endophytic bacteria and reported *Bacillus pumilus* to be a dominating species among 18 different types of bacteria. In another study, Huang et al. (2015) reported the presence of distinct bacterial community in agarwood and non-agarwood plant of *A. sinensis*. It has been reported that local tree environment and wound microclimate also affect the succession pattern of the fungal population (Mohamed et al., 2014). The community of bacterial endophytes are also affected by the formation of agarwood and on metabolic changes in the plant (Huang et al., 2015). It is important to compare the microbial community structure and their association with the artificially induced agarwood formation at different locations for determining responsible microbes in artificial infection.

The natural development of agarwood takes 25–30 years and the yield is less, thus it unable to meet the demand of the growing market (Zhang et al., 2010). The increasing commercial demand has led to forced development of artificial infection methods. Different methods, such as physical, chemical, and biological or combinations have been reported for artificially inducing resinous oil in *Aquilaria* tree (Ito et al., 2005; Pojanagaroon and Kaewrak, 2005; Gong and Guo, 2009; Okudera and Ito, 2009; Kumeta and Ito, 2010; Novriyanti et al., 2010; Chen et al., 2011; Wei et al., 2012; Liu et al., 2013). The physical methods include severe bark removal, inflicting nail and axe wounds, making hole with screws, inflicting wounds with chisels, partial-trunk pruning, burning-chisel-drilling, pin-hole, and agar wit are recently reported (Pojanagaroon and Kaewrak, 2005; Liu et al., 2013; Tian et al., 2013). The biological methods include fungi-based inoculation, introduced by Tunstall (Gibson, 1977). Few fungal isolates, such as *Melanotus flavolivens, Phytium sp., Penicillium sp., Diplodia sp., Botryodyplodis sp., Lasiodiplodis sp*., and *Fusarium sp*. A2, have been used for artificial infection (Gong and Guo, 2009; Zhang et al., 2014). The role of bacteria in artificial inoculation is less explored in comparison to fungi. More research is required to look at microbial strain or consortia that can develop high yield of agarwood by artificial infection. In the present study, microbial diversity residing in *A. malaccensis* tree and surrounding soil of *A. malaccensis* growing in north-eastern region of India was explored and their potential for inducing agarwood production by artificial inoculation method was assessed.

## Materials and methods

### Sample collection and isolation of microbes

Fifty healthy trees from 21 different sites (Figure S1) in Assam, India were identified by the State Forest Research Institute, Itanagar. Stem and soil samples were collected from these sites and transported to the lab in an icebox. Isolation of microbes was done within 48 h of the collection. List of collected samples is provided at Table S1. The isolation of stem endophytes was done using surface sterilization according to the method established in our laboratory (Kumar et al., 2011) and the soil microbes were isolated by serial dilution method according to protocol of Kasana et al. (2008). Grouping of these isolates was done on the basis of morphological similarities and agar block of axenic cultures were stored in 15% glycerol stock at −70°C for long-time preservation.

### Extraction of DNA, PCR, and sequencing

The genomic DNA of pure fungal isolates was extracted using DNeasy Plant mini kit (Qiagen GmbH, Hilden Cat. No 69104) by following the manufacture's instruction. Bacterial DNA was isolated using the CTAB method (Nishiguchi et al., 2002). The extracted DNA was used for PCR amplification by primer ITS1 and ITS4 according to Kumar et al. (2011) for fungi and 518F and 1492 R universal primers for bacterial sequence. The Polymerase chain reaction was achieved in 25 μL of reaction mixture, which contained 2.5 μL of 10× PCR Buffer with 15 mM MgCl_2_ (Applied Biosystem, India), 0.5 μL of dNTP mix (10 mM, Applied Biosystem), 2.5 μL of ITS1, ITS4 for fungi, and 518F and 1492 R primers for bacteria (10 picomole/μL), 1 μL of DNA template, and 0.5 μL of Ampli Teg Gold (5 U/μL). The ITS1, ITS4, 518F, and 1492 R primers were synthesized from Merck nucleotide synthesis services, Bengaluru, India, according ITS1 5′TCCGTAGGTGAACCTGCGG3′, ITS4 5′TCCTCCGCTTATTGATATGC3′, 518F 5′-CCAGCAGCCGCGGTAAT-3′, and 1492R 5′-GGYTACCTTGTTACGACTT-3′ sequence. The Polymerase Chain Reaction was done on Veriti Thermal Cycler (Applied Biosystem) using following programmes: initial denaturation at 94°C for 2 min; 30 cycles of denaturation, annealing, and elongation at 94°C for 1 minute; 57°C for 1.30 min, and 72°C for 2 min followed by final elongation at 72°C for 4 min. The negative control was also run using sterile water. The amplified product was checked on 1.5% agarose gel by gel electrophoresis. The amplified product was sequenced by Merck sequencing services, Bengaluru, India.

### Identification of microbes and phylogenetic evaluation

The sequences were aligned and trimmed by DNA Baser 4.2 software on the basis of quality read values. The identification of microbes was done on the basis of similarity of amplified sequence with NCBI database using Basic Local Alignment Search Tool (nBLAST) of the US National Centre for Biotechnology Information (NCBI), SILVA and UNITE databases. The sequences were submitted to NCBI database. The phylogenetic tree was developed using Neighbor-Joining and Maximum Likelihood method by MEGA 6.0 software using sequence alignment by Clustal W pairwise sequence Alignment tool of the EMBL Nucleotide Sequence Database. The evolutionary distances were computed using the Maximum Composite Likelihood method.

### Evaluation of microbial diversity

The microbial diversity in the stem and surrounding soil of *A. malaccensis* was determined by the Shannon diversity index, Simpson's index, and Fisher Alpha index. Species richness of the isolated microbes was estimated according to the Menhinick's index (D*mn*) by the following equation (Whittaker, 1972):

(1) $$\begin{array}{r} Dmn=\frac{s}{\sqrt{N}} \end{array}$$

Where “s” represents the number of different type of species in a sample and “*N”* represents the total number of isolates in a given sample. Simultaneously, dominance of class was also determined by Camargo's index (1/*Dmn*), where “*Dmn”* denotes species richness. A species was explained as dominant if *Pi* > 1/ *Dmn*, where *Pi* represents the relative abundance of a species, *i* explains the total number of competing species in the community (Camargo, 1992). The diversity was also evaluated to understand the distribution of organisms as randomly, aggregated, or uniformly distributed. Shannon diversity index (*H*′), Simpson's index, and Fisher Alpha index were calculated by SPADE programme version 3.1 (Chao and Shen, 2010). The heat map analysis was also done for soil and stem isolates using online tool Heatmapper^1^ (Babicki et al., 2016). The pairwise heat map was generated by average linkage calculation using Euclidean distance measurement method.

### Agarwood formation by artificial infection method

Forty microbial isolates selected on the basis of their dominance were screened for their potential to induce agarwood formation by artificial infecting the wood with these micobes. For this, fungal mycelium was grown on PDA media and 5 days old fungal plugs were transferred to 100 ml of potato dextrose broth. The cultures were incubated at 25°C for 7 days. While, bacterial cultures were grown in 100 ml of nutrient broth medium and incubated for 48 h at 37°C. The grown fungal biomass was collected by filtration with Whatman filter paper no 1 and bacterial biomass with centrifugation at 8,000 rpm for 10 min and lyophilized to get powder form. The lyophilized sample was transported in sterile falcon tubes at experimental site. The inoculum was developed in 10 ml of 2% glucose solution. The artificial infection was done in *A. malaccensis* tree in Dergaon, Assam using syringe method. Artificial infection was introduced in 4–5 years old *A. malaccensis* trees. Holes were made in a zigzag manner with a drill machine (5 mm diameter bit size) in the trunk of the tree. The initial hole was made 20 cm above from the ground and next wound was drilled at 10 cm interval and the depth of the drill was 1.5 cm. Around 1 ml of homogenized microbial culture was inoculated in each hole using sterile syringe and hole was covered with para film. Each strain was infected in replicates. A sterilized medium was used as a syringe control.

### Gas chromatography–mass spectroscopy analysis

The wood samples (~2–3 g) were collected 3 months after inducing artificial infection by drilling the wood around the inoculated hole. The collected of infected wood dust sample (1 g) were extracted in ethyl acetate by the reflux method. Uninoculated wood dust (wood control) and media control wood dust (syringe control) were also treated in similar manner to compare chemical composition with treated wood dust samples. The extract was concentrated in rotary evaporator. The concentrated extract was dissolved in 1 mL of dichloromethane and analyzed by Gas Chromatography-Mass Spectroscopy (GC-MS) (7890A/5975C, Agilent, California, United States) using an DB-WAX capillary column (30 m × 0.25 mm; film thickness 0.25 μm) and using Helium (purity > 99.999%) as the carrier gas with the constant flow rate of 1.0 mL/min. Around 1 μL of injection volume was used. The temperature of injector part was 230°C and oven temperature programing was used. The initial oven temperature was maintained at 80°C for 1 minute, then increased to 150°C @ 10°C/min in 7 min followed by temperature increase to 250°C @ 5°C/min till 22.5 min. The Mass Spectroscopic system was operated in EI mode (70 eV). Mass of the compounds was analyzed in the range of m/z 50–500 amu.

### Data analysis

Unscrambler version 10 (CAMO, USA) was used to perform Principal Component Analysis (PCA) and statistical analysis.

## Results

### Identification of fungi and bacteria and their distribution

**Figure 1:**
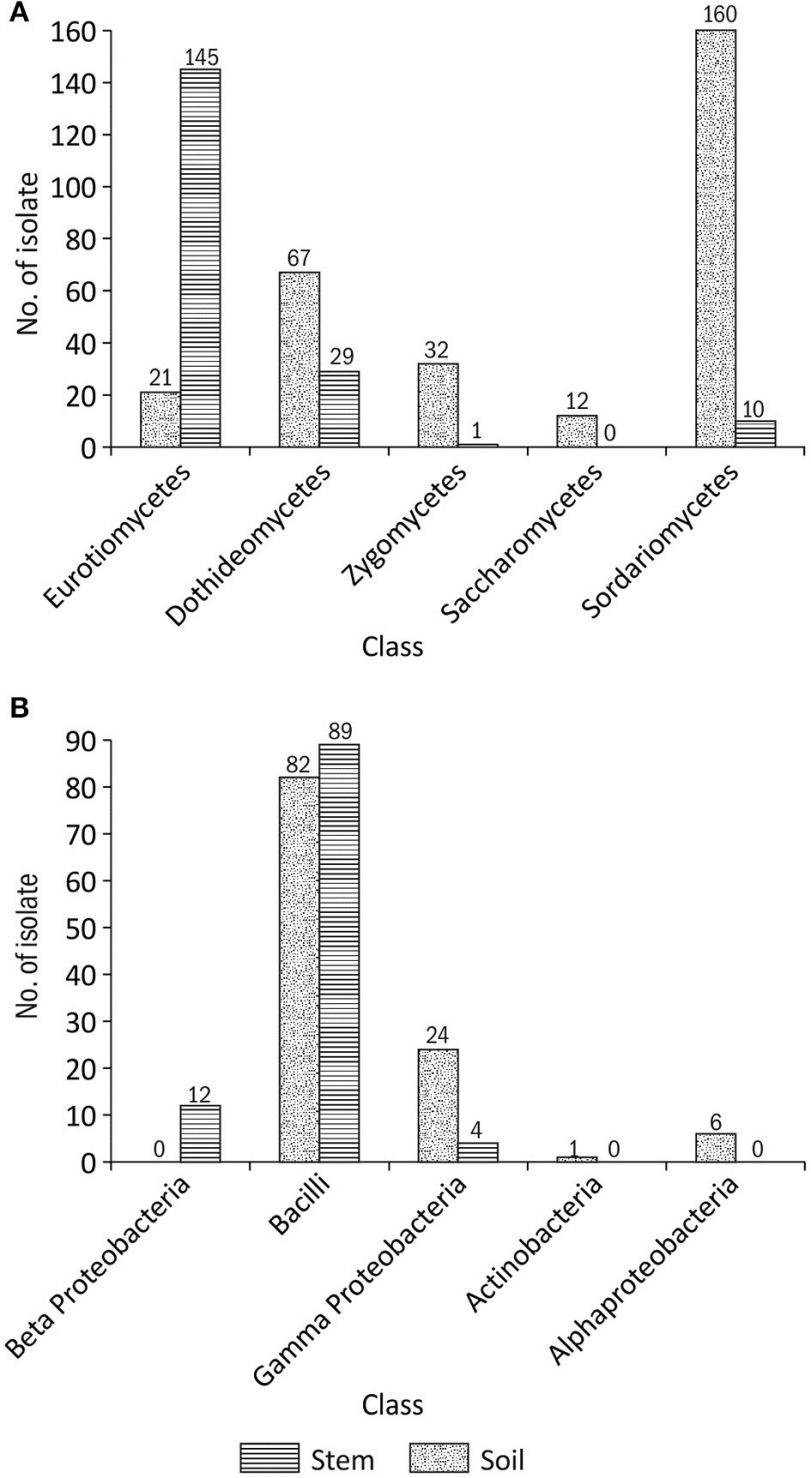
Diversity of fungal **(A)** and bacterial **(B)** isolates in the stem and soil samples of *Aquilaria malaccensis*.

**Figure 2:**
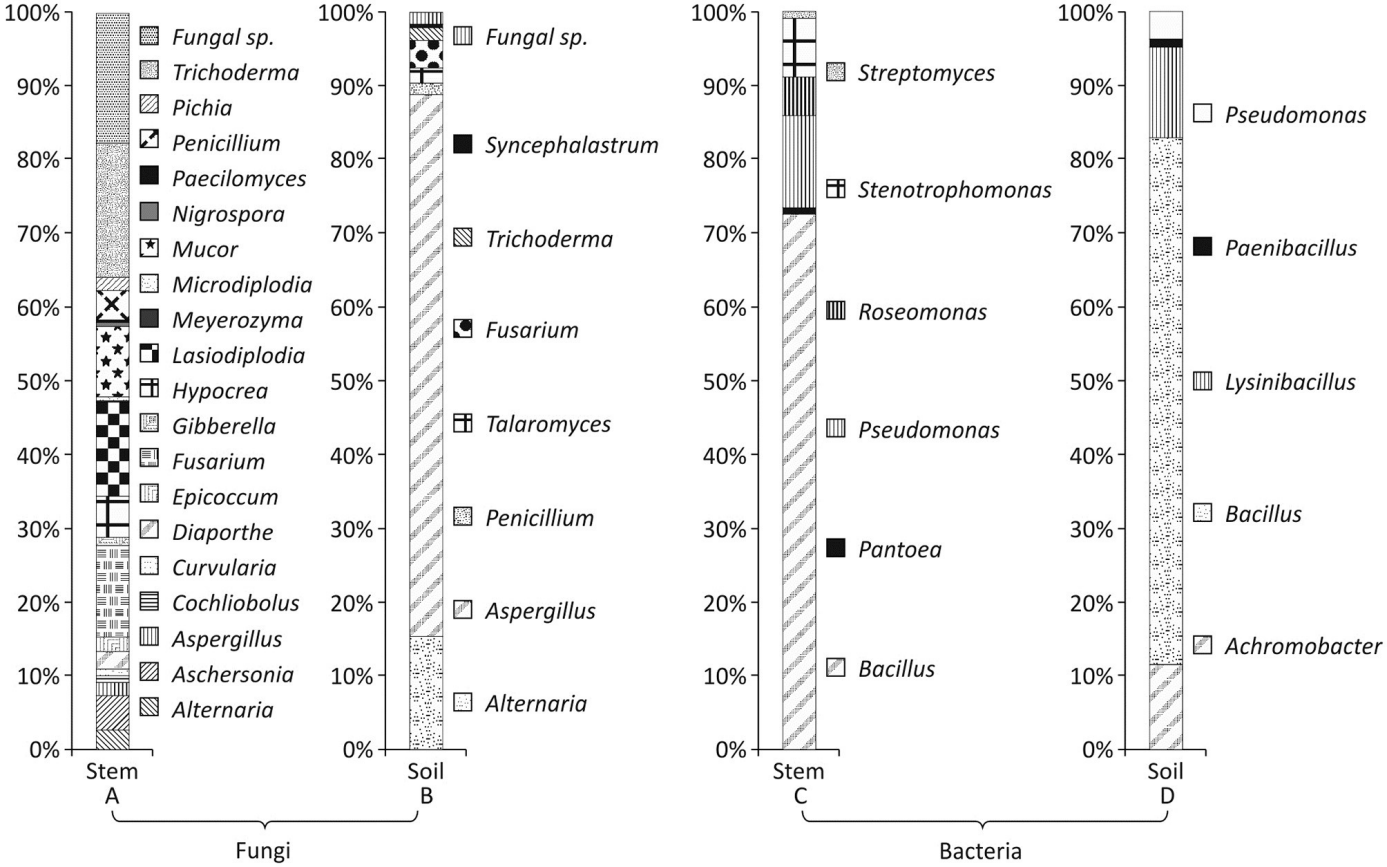
Distribution of fungi **(A,B)** and bacteria **(C,D)** in stem and soil samples at genus level.

In all 340 fungi and 131 bacteria were isolated from 50 stem samples, and 188 fungi and 148 bacteria were isolated from 50 soil samples. These isolates were grouped on the basis of similarity of their external morphology and microscopic examination and representative isolates were used for molecular identification by 16s rRNA region amplification using 518F and 1492 R primers for bacteria and ITS region amplification using ITS1 and ITS4 primers for fungi and sequenced using Merck sequencing services, Bengaluru, India. Distribution of fungi and bacteria up to class level is given in Figure 1 and up to genus level in Figure 2. The list of identified strains and their accession number along with % similarity with the best match with different sequence databases is given in supplementary information Table S2. The identification based on ITS sequence of fungal strains showed maximum diversity in the stem samples (Table **3**). It was observed that in the stem five fungal classes viz. Eurotiomycetes, Dothideomycetes, Zygomycetes, Saccharomycetes, and Sordariomycetes represented fungal community, while in the soil samples only four out of the five classes were present. Saccharomycetes were not isolated from stem samples. In the case of bacteria Bacilli class was dominant in the stem and soil samples.Actionbacteria and Alpha Proteobacteria members were obtained only from the stem and Beta Proteobacteria only from the soil samples.

### Phylogenetic and microbial diversity analysis

**Figure 3:**
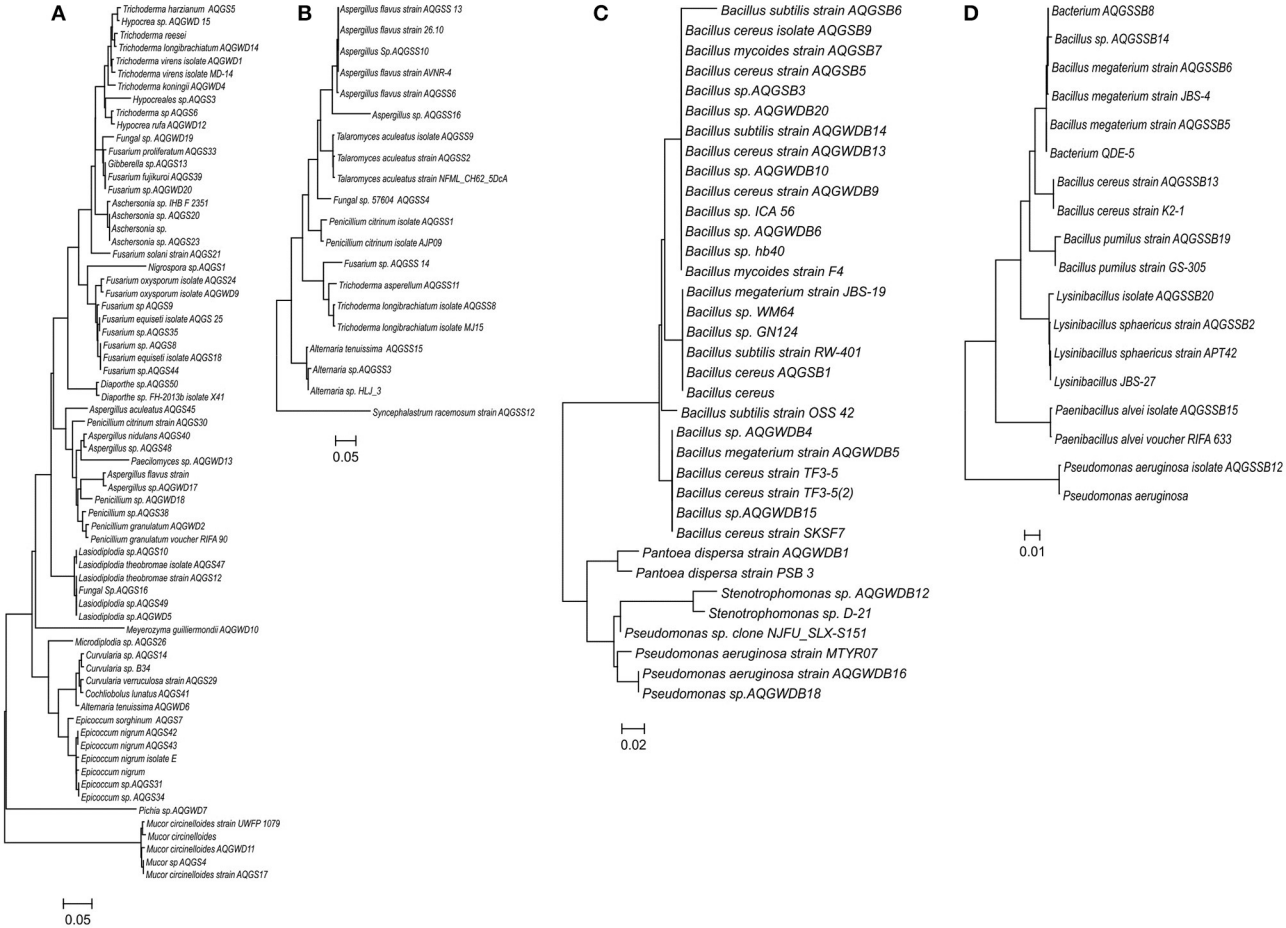
Phylogenetic trees based on Neighbor-Joining method of the r DNA and ITS sequence of different fungi from stem **(A)** and soil **(B)** and bacterial isolates from stem of *Aquilaria malaccensis* **(C)** and soil **(D)**. 1000 replicates were calculated in the bootstrap.

**Figure 4:**
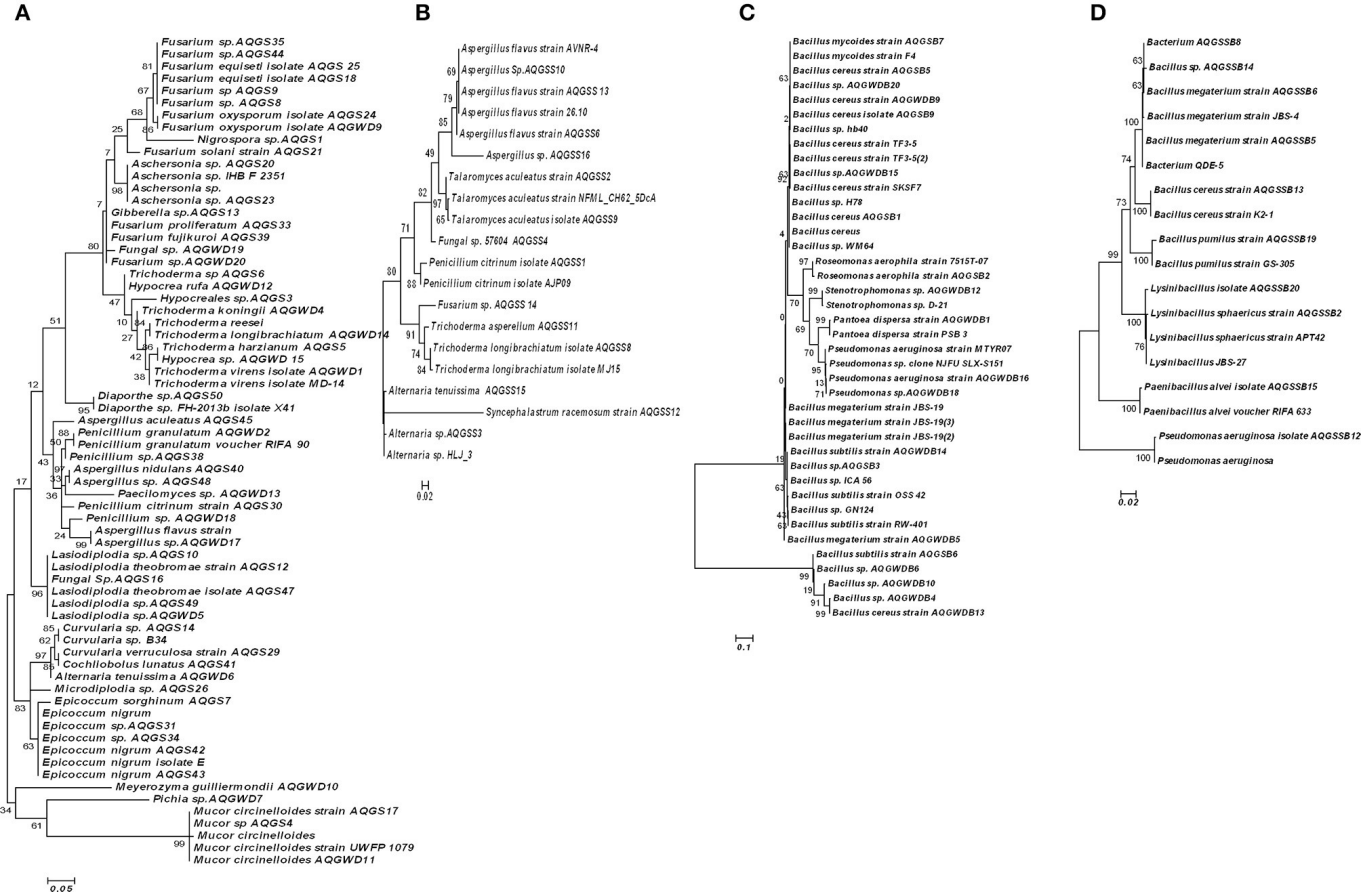
Phylogenetic trees based on Maximum likelihood method of the SSU ribosomal sequence of different fungi from stem **(A)** and soil **(B)** and bacterial isolates from stem of *Aquilaria malaccensis* **(C)** and soil **(D)**.

The phylogenetic relation between different morphotypes isolated from stem and soil samples were measured by MEGA version 6.06 using the Neighbor-Joining (N-J) and Maximum Likelihood (M-L) method (Figures 3, 4). Both type of method showed similar type of fungal and bacterial clusters. But in stem fungal isolate, *Meyerozyma guilliermondii* AQGWD10 showed less distant from *Lasiodiplodia* sp. cluster in N-J method while, in M-L method *M. guilliermondii* showed close to *Pichia* sp. (Figure 3A). In the case of soil fungal, soil and stem bacterial isolates cluster pattern was similar in both N-J and M-L method. In stem, 19 genera from 11 fungal families were identified using sequence similarity with NCBI database. In the stem, Hypocreaceae family was most dominant contributing 23.8% to richness while *Hypocrea* and *Trichoderma* genera, Botryospaeriaceae and Nectriaceae were second most commonly occurring family with 13.2 and 13.5% richness, having *Microdiplodia, Lasiodiplodia, Fusarium*, and *Gibberella* genera (Table 1). Pleosporace and Trichocomaceae family contributed only 6.5 and 6.2% richness, but contained 4 and 3 types of genera, including *Cochliobolus, Curvularia, Alterneria*, and *Epicoocum* in Pleosporace family and *Aspergillus, Penicillium*, and *Paecilomyces* in Trichocomaceae family. Around 14.1% of unidentified fungal sp. also contributed in fungal diversity. Likewise, in soil samples Trichocomaceae family showed the highest with 77.1% richness and members of Syncephalastraceae family was only isolated from soil samples.

**Table 1:** Family dominance of fungus and bacteria isolated from stems and soil samples of *A. malaccensis*.

**S. No**.	**Fungal family**	**Richness (%)**		**Bacterial family**	**Richness (%)**	
		**Stem**	**Soil**		**Stem**	**Soil**
1	Botryosphaeriaceae	13.20	0.0	Acetobacteraceae	4.6	0.0
2	Clavicipitaceae	04.70	0.0	Alcaligenaceae	0.0	8.11
3	Debaryomycetaceae	00.30	0.0	Bacillaceae	62.6	59.5
4	Diaporthaceae	02.40	0.0	Enterobacteriaceae	0.8	0.0
5	Fungal sp	17.60	1.6	Paenibacillaceae	0.0	0.68
6	Hypocreaceae	23.80	1.6	Pseudomonadaceae	10.7	2.7
7	Mucoraceae	09.40	0.0	Xanthomonadaceae	6.9	0.0
8	Nectriaceae	13.50	3.7			
9	Pleosporaceae	06.50	15.4			
10	Saccharomycetaceae	01.80	0.0			
11	Syncephalastraceae	00.00	0.5			
12	Trichocomaceae	06.20	77.1			
13	Trichosphaeriaceae	00.60	0.0			

The phylogenetic relation of these isolates is given in the Figures 3, 4. In the case of bacteria, Bacillaceae is the dominant family in the stem and soil with 62.6 and 59.5% richness, respectively. Various bacterial families were isolated only in stem samples. Xanthomonadaceae showed 10.7% dominance, followed by Acetobacteraceae (4.6%) and Enterobacteriaceae (0.8%), while Alcaligenaceae and Paenibacillaceae showed presence only in the soil samples with 8.11% and 0.7% richness. Debaryomycetaceae, Saccharomycetaceae, Diaporthaceae, Clavicipitaceae, and Trichosphaeriaceae fungal families were exclusively isolated from stem, while Syncephalastraceae from soil samples. Similarly in bacteria, Enterobacteriaceae, Acetobacteraceae, and Xanthomonadaceae family was exclusively isolated from stem.

**Figure 5:**
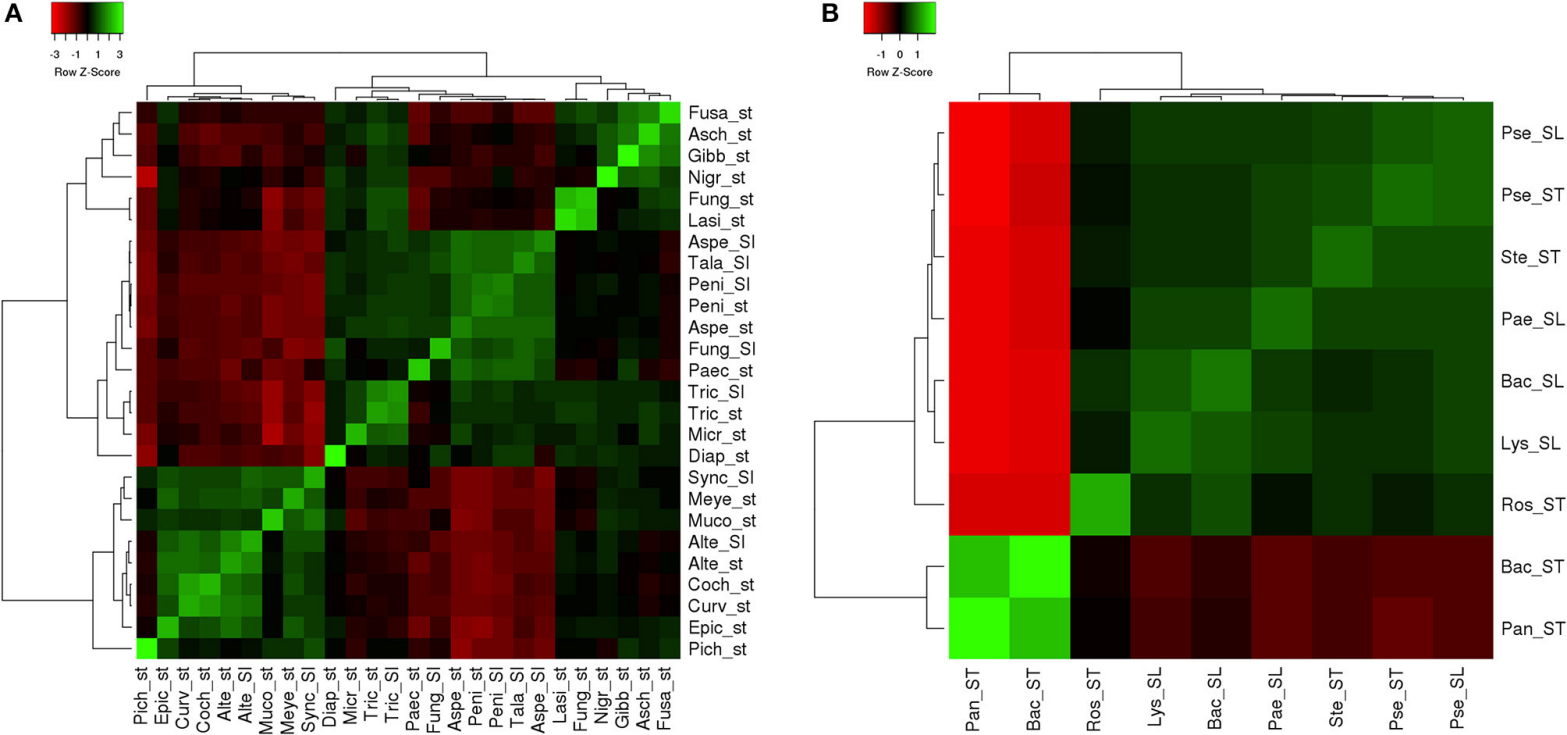
Heat map analysis of **(A)** fungi and **(B)** bacterial isolates from stem and soil samples. St, stem isolate; Sl, Soil isolate; Pich, *Pichia*; Epic, *Epicoccum*; Curv, *Curvularia*; Coch, *Cochliobolus*; Alte, *Alternaria*; Muco, *Mucor*; Meye, *Meyerozyma*; Sync, *Syncephalastrum*; Diap, *Diaporthe*; Micr, *Microdiplodia*; Tric, *Trichoderma*; Pace, *Paecilomyces*; Fung, Fungal; Aspe, *Aspergillus*; Peni, *Penicillium*; Tala, *Talaromyces*; Lasi, *Lasiodiplodia*; Nigr, *Nigrospora*; Gibb, *Gibberella*; Asch, *Aschersonia*; Fusa, *Fusarium*; Pan, *Pantoea*; Bac, *Bacillus*; Ros, Roseomonas; Lys, *Lysinibacillus*; Pae, *Paenibacillus*; Ste, *Stenotrophomonas*; Pse, *Pseudomonas*.

The fungal diversity of *A. malaccensis* in the North Eastern part of India was measured using Shannon diversity index (*H*′), Simpson index (D), and Fisher alpha index (α). Species richness in each source was measured by the Menhinick's diversity index (*Dmn*). The diversity index for all samples were measured by SPADES software and presented in Table 2. It was observed that stem were rich in fungal species (*Dmn* = 0.034) followed by soil (*Dmn* = 0.02). A similar pattern was measured in bacteria also (stem = 0.03 and soil = 0.02). Source-specific fungal dominance by Camargo's index was the highest measured in soil (47.00) followed by stem (29.57) and bacterial dominance also observed the highest in soil (49.33) than stem (37.43). The dominant genus was *Tricoderma* in stem with their *Pi* = 0.18, while in soil *Aspergillus* showed dominance with *Pi* = 0.73. The next dominance genus was *Lasiodiplodia* (*Pi* = 0.13) and *Fusarium* (*Pi* = 0.12) in stem and *Alternaria* (*Pi* = 0.15) in soil. Rests of fungal species were less dominant <0.09. In bacteria, Bacillus genera showed dominance in both root and soil samples with *Pi* = 0.62 and 0.51, respectively. *Pseudomonas* was dominant in the stem and *Lysinibacillus* in soil with *Pi* = 0.10 and 0.09, respectively. The Pielou's evenness index showed the distribution of fungal and bacterial species evenly in soil (*J*′ = 0.18 and 0.25) and stem (*J*′ = 0.42 and 0.25). The Maximum Shannon diversity index was observed for stem fungal isolates (*H*′ = 2.43) accompanied by soil bacteria (*H*′ = 1.25), while the Simpson diversity index was the highest in soil bacteria (*H*′ = 2. 84) followed by stem bacteria in comparison to fungi. Fisher alpha (α) was measured the highest in fungal stem (α = 5.57), followed by fungal soil (α = 1.70) samples. The similarity of fungal and bacterial isolates from stem and soil were done using heat map expression. It is observed that stem and bacterial isolates showed less variation at genera level and creating a cluster in heat map (Figure 5).

**Table 2:** Diversity indexes of fungi and bacteria in the different samples.

**Diversity index**	**Fungi**		**Bacteria**	
	**Stem**	**Soil**	**Stem**	**Soil**
Shannon (*H*')	2.43	0.95	1.20	1.30
Simpson index (*D*)	0.10	0.56	0.42	0.40
Simpson Diversity index (1-D)	0.90	0.44	2.35	2.80
Fisher (α)	5.57	1.70	1.58	1.30
Menhinick's Diversity (*Dmn*)	0.034	0.02	0.03	0.02
Pielou's Evenness index (*J*')	0.42	0.18	0.25	0.25
Camargo's index (1/ *Dmn*)	29.57	47.0	37.43	49.33

### Induction of artificial infection

**Figure 6:**
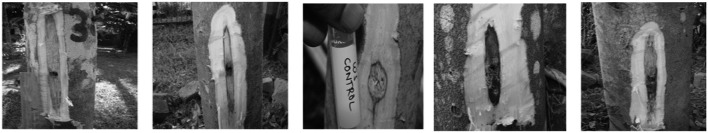
Artificial infection in different *Aquilaria malaccensis* trees after 3 months of inoculation of fungal and bacterial strain: Dark brownish color of wood showing the initiation of artificial infection.

Total 21 fungal and 19 bacterial strains isolated from infectious stem and surrounding soil were screened to assess their potential for induction of artificial infection in *A. malaccensis* tree. It was observed that the color of wood changed from white to brown/black after 3 months of infection in infected trees (Figure 6). Wood dust was harvested after 3months of inoculation and subjected to GC-MS analysis for confirmation of artificial infection on the basis of production of compounds presented in agarwood oil. In our previous study on comparison of GC-MS profile of infected and uninfected woods, we identified agarospirol, benzyl acetone and anisyl acetone as marker compounds for infection therefore, these compounds were selected as indicator for agrawood formation. Presence of agarospirol compound in the harvested wood confirmed the initiation of infection in the tree (Figure S2 and Table 3). Other details such as percentage of benzyl acetone and anisyl acetone are provided in Table S3.

**Table 3:** Agarospirol content and infection length obtained with different fungal and bacterial strains 3 months after the artificial inoculation in the *A. malaccensis* trees.

**S. No**.	**Species**	**Type of organism**	**Source**	Agarospirol^*a,b,c^ (2-(6,10-Dimethylspiro[4,5]dec-6-en-2-yl)-2-propanol)	**Infection**
				**Content (%)**	**Length (cm)**
1	*Pantoea dispersa* AQGWDB1	Bacteria	Stem	3.77	1.26
2	*Penicillium polonicum AQGGR1.1*	Fungi	Stem	3.33	2.60
3	*Syncephalastrum racemosum AQGSS 12*	Fungi	Soil	1.34	2.60
4	*Penicillium aethiopicum AQGGR1.2*	Fungi	Stem	0.67	3.00
5	*Trichoderma asperellum AQGSS 11*	Fungi	Soil	0.20	3.00
6	*Bacillus cereus AQGWDB9*	Bacteria	Stem	0.09	3.45
7	*Penicillium polonicum AQGGR1.1*	Fungi	Stem	0.08	2.60
8	(Unidentified) AQGSSB23	Bacteria	Soil	0.06	2.00
9	*Bacillus anthricus strain AQGWDB2*	Bacteria	Stem	0.05	2.91
10	*Rhizopus oryzae AQGGR1.3*	Fungi	Stem	0.05	2.00
11	(Unidentified) AQGWDB7	Bacteria	Stem	0.05	3.33
12	*Bacillus megaterium AQGWDB 3*	Bacteria	Stem	0.03	2.35
13	Wood control	Control		0.00	0.00

* *Variance comparison at significant level: 0.05*.

*a *Fungi vs. control- P-value < 0.001*.

*b *Bacteria vs. control -P-value < 0.001*.

*c *Bacteria vs. Fungi -P-value = 0.0057231*.

It was noted that in comparison to uninfected wood and experimental control (syringe and wood controls) agarospirol was detected only in artificially infected wood samples. It is reported that the presence of agarospirol is one of the responsible compound for fragrance in agarwood (Kalra and Kaushik, 2017). Highest agarospirol content was measured in wood infected by *Pantoea dispersa* (3.77%), followed by fungi *Penicillium polonicum* (3.33%), *Syncephalastrum racemosum, Penicillium aethiopicum, and Trichoderma asperellum* (Table 3). The infection length was measured in the range of 2.00 to 11.83 cm. Strain *Paenibacillus alvei* AQGSSB15 showed the highest infection up to 11.83 cm in length. Although, agarospirol could not be detected in this sample, however, it showed presence of benzyl acetone confirming early stage of infection (Table S3). Highest agarospirol was measured in the sample infected by *Pantoea dispersa* AQGWDB1 and length of infection was found only up to 1.26 cm. Therefore, no relation was observed between infection length and agarospirol production. Based on the agarospirol formation, 31% of bacterial and 23% of fungal isolates showed their ability to produce agarospirol in agarwood through artificial infection (Table 3). Production of agarospirol was significantly higher with bacterial inoculum than the fungal inoculum with *P*-value 0.0057 (Figure S3). Principal component analysis (PCA) also showed two distinct clusters of bacteria and fungi (Figures S4, S5).

## Discussion

### Fungi and bacteria identification and their distribution

Endophytes play an important role in plant physiology. Most of the endophytes are helpful in host plant growth, stress tolerance, and explained as the microorganisms that are not detrimental to the plant (Oses et al., 2008; Huang et al., 2009; Nimnoi et al., 2010). However, some had no beneficial impact and showed latent pathogenicity in plant species (Pojanagaroon and Kaewrak, 2005). Latent pathogenicity of endophytes could be an economical source of local community and *Aquilaria* is a live example in this regard, which produces oleoresin after infection and enhances the value of tree in market (Barden et al., 2000; Pojanagaroon and Kaewrak, 2005; Chhipa et al., 2017). Various studies have been done on isolation of endophytes from *Aquilaria* species, such as Malyasian *A. malaccensis* (Mohamed et al., 2010; Bhore et al., 2013); Chinese *A. sinensis* (Gong and Guo, 2009; Tian et al., 2013); Thai *A. crassna* (Nimnoi et al., 2010); *A. agallocha* (Tamuli et al., 2000), and Indian *A. malaccensis* (Bhore et al., 2013). However, very few reports are available on the development of artificial infection by using these endophytes (Mitra and Gogol, 2001; Mohamed et al., 2014). In the present study endophytic microbes of *Aquilaria* tree, and soil microbes isolated from circumventing soils of *Aquilaria* tree were successfully utilized for induction of agarospirol formation through artificial inoculation,. *Trichoderma virens* was found as the most abundant fungi in stem followed by *Lasiodiplodia theobromae, Lasiodiplodia* sp., and *Fusarium equiseti* (4.9%). While Mohamed et al. (2010) observed a reverse pattern with *Fusarium* sp. as the most dominant and *Trichoderma* sp. as the least dominant in infected and similar fungal strains in non-infected samples of *A. malaccensis* in Malaysia region and suggested that these fungi might have little role in resin formation. Similarly, *Colletotrichum, Botryosphaeria, Phomopsis, Gloeosporioides*, and *Cylindrocladium* species are reported in wounded *Aquilaria* tree, while *Alternaria, Mycosphaerella, Ramichloridium, Sagenomella*, and *Phoma* species is in non-resinous tree of *A. sinensis* in China (Tian et al., 2013). While in the case of bacteria dominance of *Bacillus*, genus was observed in both soil and stem samples. *Pantoea, Roseomonas, Stenotrophomonas*, and *Streptomyces* genera were only obtained from stem samples, while *Achromobacter, Lysinibacillus*, and *Paenibacillus* were only observed in soil samples. Presence of endophytic actinomycetes including *Streptomyces, Nonomuraea, Actinomadura, Pesudonocardi*, and *Nocardia* genera is also reported in *A. crassna* (Nimnoi et al., 2010). Similarly, Bhore et al. (2013) demonstrated dominance of *Bacillus pumilus* (36.4%) among 18 different bacterial species in *Aquilaria* sp. of Malaysia region.

### Phylogenetic and microbial diversity analysis

In the case of the *Aquilaria* plant, microbial diversity and dominance could be species and region specific. Kusari et al. (2012) observed that 70% of isolates in soil and plant part are common. It suggests that the plant and endophytes association developed after overcoming many physical and chemical barriers and particular fungi established as endophytes in a particular niche or localized in the tissue in a systemic manner (Hyde and Soytong, 2008). Subsequently, pathogenicity is also affected by the surrounding environment as endophytic to host plant in one ecosystem can be pathogenic in another ecological niche. It is reported that endophytic and pathogenic lifestyle are inter convertible due to environmental, chemical, and molecular elicitors (Schulz et al., 2002; Eaton et al., 2011).

In the present study, higher diversity of microbial isolates has been observed in the stem than the soil samples. The Shannon and Fisher alpha diversity index in the stem measured the highest value showing diversity and abundance of endophytes in stem comparison to soil samples. The results are similar to previously reported by Kumar and Hyde (2004) in *Tripterigium wilfordii* and Gond et al. (2012) in *Nyctanthes arbortristis* showed the highest Shannon index in stem.

### Induction of artificial infection

The development of artificial infection method using endophytic and soil microorganisms will provide significant impact on agarwood production. In present study, 31% of bacterial and 23% of fungal isolates showed positive response in agarwood formation by production of agarospirol compound within 3 months of infection. To the best of our knowledge no previous study has reported agarospirol production by artificial infection in 3 months. Zhang et al. (2012) reported agarwood production in 6 months after infection, while Lin et al. (2010) after 12 months. The maximum amount of Agarospirol was induced by *Pantoea dispersa* AQGWDB1 followed by *Penicillium polonicum* AQGGR1.1. Previously, various researchers have used fungi in Agarwood formation (Chhipa et al., 2017). However, this is the first report on use of bacteria in artificial infection and agarospirol production (Bose, 1934; Bhattacharyya et al., 1952; Jalaluddin, 1977; Venkataramanan et al., 1985; Beniwal, 1989; Tamuli et al., 2000; Mitra and Gogol, 2001). Further, Studies on artificial infection have been reported by *Aspergillus* sp., *Lasiodiplodia* sp., *Fusarium* sp., *Penicillium* sp., and *Trichoderma* sp. (Yunita, 2009; Akter et al., 2013). Recently, Mohamed et al. (2014) also reported artificial Agarwood formation in young *A. malaccensis* tree by fungal inoculation. Similarly, Lin et al. (2010) also reported that fungus *Melanotus flavolivence* is also capable of inducing Agarwood formation after 6 months of infection. It has been reported that the accumulation of plant secondary metabolites can be the result of microbial stimulation. Cui et al. (2013) also demonstrated the production of resin-containing organic volatile fatty acid in response to fungal attack. This process is called “tylosis.” The resin increases the density and changes the color of the wood from pale to dark brown or black. In present study, it was observed that 41% of stem isolates showed their potential in development of agarospirol while 13.6% soil isolates could induce agarwood infection, confirming the role of endophytes in agarwood formation. This study is extremely beneficial for local tribes of north-eastern part of India where Agarwood is treated as an economic resource (Lin et al., 2010). Identification of such microbes that can induce agarwood production at short time exposure and validation at field levels has lot of potentials for future research.

## Conclusion

Endophytes are helpful in plant physiology by producing different compounds, which assist in plant growth, stress tolerance, and plant immunity. Isolation and identification of such microbes are an important aspect for biotechnological applications. In present study, we could isolate total 528 fungi and 279 bacteria from Aquilaria stem and surrounding soil samples collected from different sites of Assam. Bacilli were found most dominant class in the bacteria and Sordariomycetes in fungi. In stem, Hypocreaceae was measured most dominant fungal family with 23.8% richness while Bacileaceae was dominant bacterial family with 62.6 and 59.5% richness in both stem and soil isolates. Maximum Shannon diversity index was measured in stem fungal isolates (H′ = 2.43). Agarospirol production was induced successfully using bacteria *Pantoea dispersa* (3.77%) and fungi *Penicillium polonicum* (3.33%) within 3 months only, after inducing the infection. The agarospirol is one of the responsible compounds for fragrance in Agarwood. The use of such microbes in artificial production of agarospirol could be source of economic development of villagers of North Eastern part of India.

## Author contributions

Conception and designed the experiments: HC and NK. Performed the experiments: HC. Analyzed the data: HC and NK. Contributed reagents/materials/analysis tools: HC and NK. Wrote and enriched the literature: HC and NK. Corrected the manuscript: NK.

### Conflict of interest statement

The authors declare that the research was conducted in the absence of any commercial or financial relationships that could be construed as a potential conflict of interest.

## Footnotes

^1^http://www.heatmapper.ca.

## Supplementary material

Figure S1 Site map of the districts selected for sample collection.

Figure S2 GC-MS profile of oil extracted from artificial infected wood dust sample inoculated with *Pantoea dispersa* AQGWDB1 **(A)** and *Penicillium aethiopicum* AQGGR1.2 **(B)** showing the presence of the agarospirol compound in gas chromatogram mass spectrum of Agarospirol compound (RT 35.72) **(C)**.

Figure S3 *F*-test of significance for agarospirol content variation between fungal infected and control wood **(A)**, bacterial infected and control wood **(B)** and fungal and bacterial infected wood **(C)**.

Figure S4 Bi plot of GC data of bacterial and fungal infected wood samples collected 3 months after artificial inoculation  Bacteria  Fungi.

Figure S5 PCA plot of GC data of bacterial and fungal infected wood samples collected 3 months after artificial inoculation  Bacteria  Fungi.

Table S1 List of sample collected from different districts of Assam for microbial isolation.

Table S2 Identification of fungal and bacterial isolates on the basis of sequence similarity with different sequence data base.

Table S3 Per cent composition of compounds in oleoresin extract identified by gas chromatography mass spectroscopy.
