# Supplementary figures and images for: Fungal and Bacterial Diversity Isolated from Aquilaria malaccensis Tree and Soil, Induces Agarospirol Formation within 3 Months after Artificial Infection
Source: Front Microbiol. 2017 Jul 11;8:1286. doi: 10.3389/fmicb.2017.01286 (PMC5507295; doi:10.3389/fmicb.2017.01286)

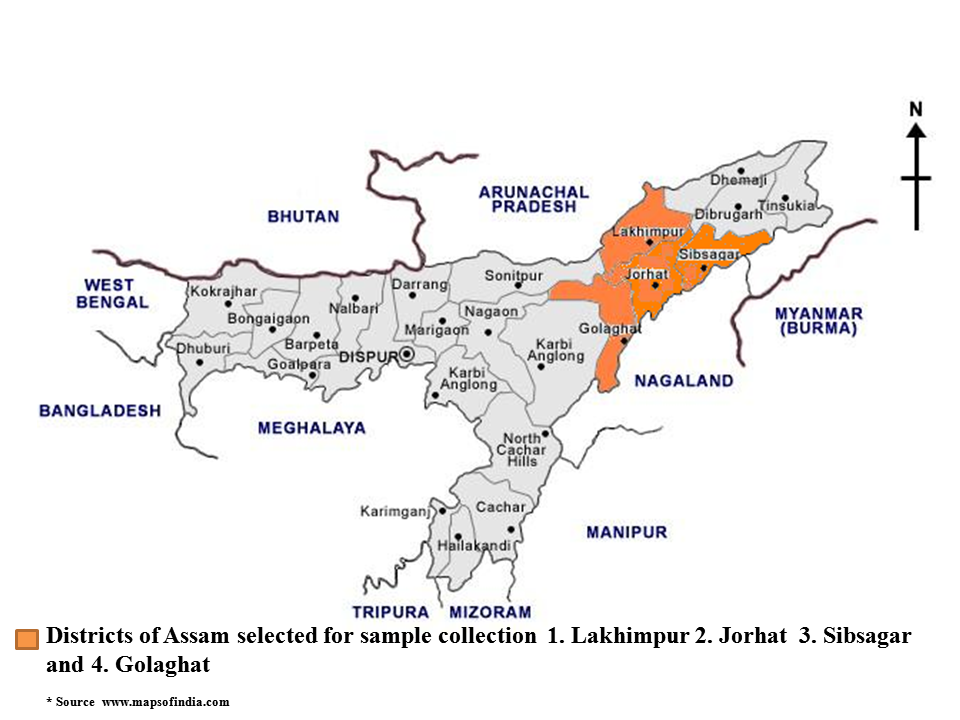

Supplement: Figure S1 — Site map of the districts selected for sample collection. [file Image1.TIF]

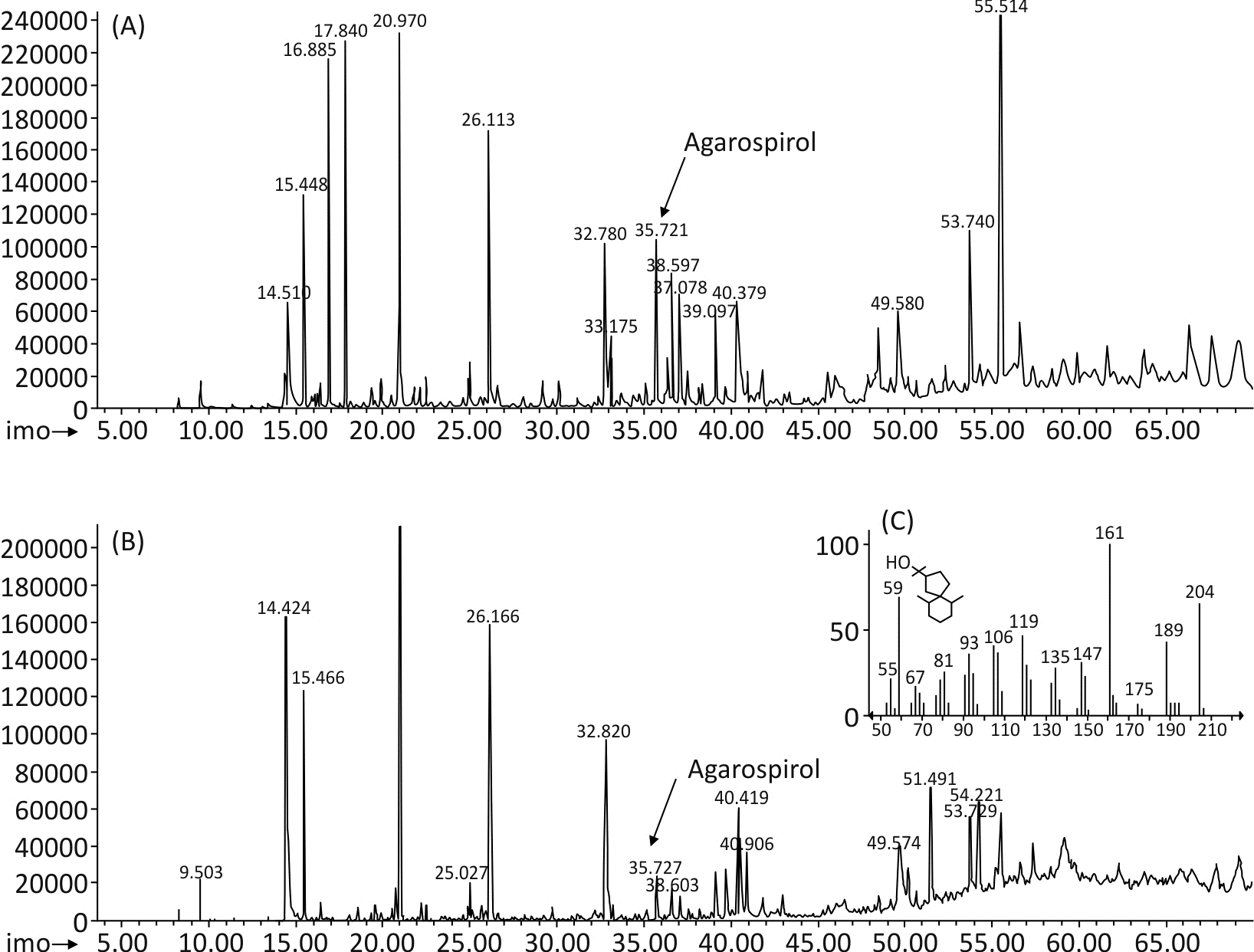

Supplement: Figure S2 — GC-MS profile of oil extracted from artificial infected wood dust sample inoculated with Pantoea dispersa AQGWDB1 (A) and Penicillium aethiopicum AQGGR1.2 (B) showing the presence of the agarospirol compound in gas chromatogram mass spectrum of Agarospirol compound (RT 35.72) (C). [file Image2.TIFF]

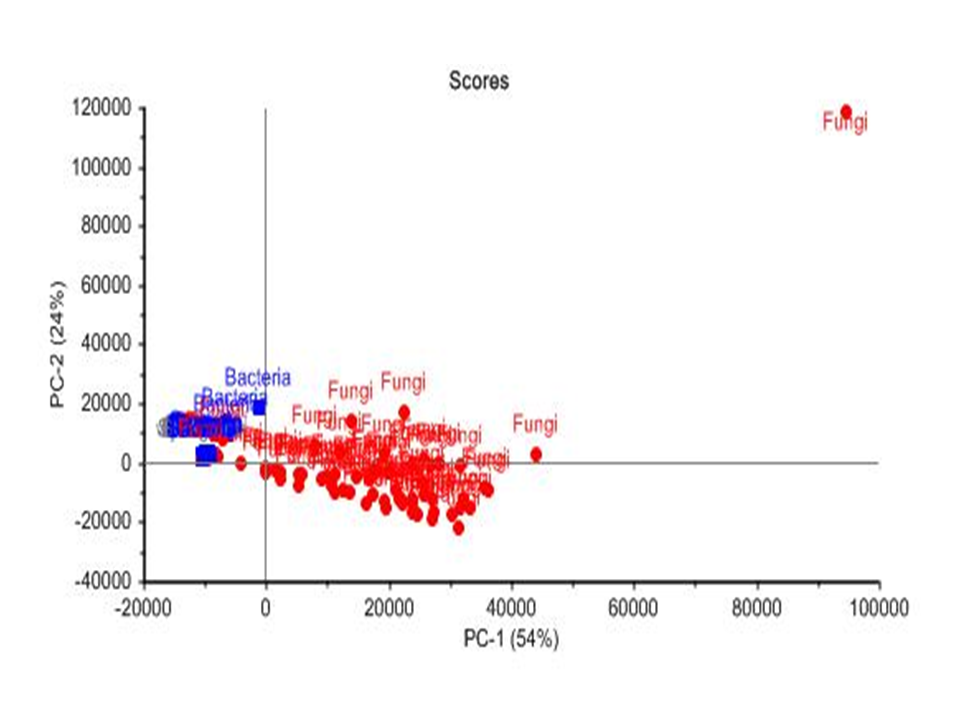

Supplement: Figure S4 — Bi plot of GC data of bacterial and fungal infected wood samples collected 3 months after artificial inoculation Bacteria Fungi. [file Image4.TIF]

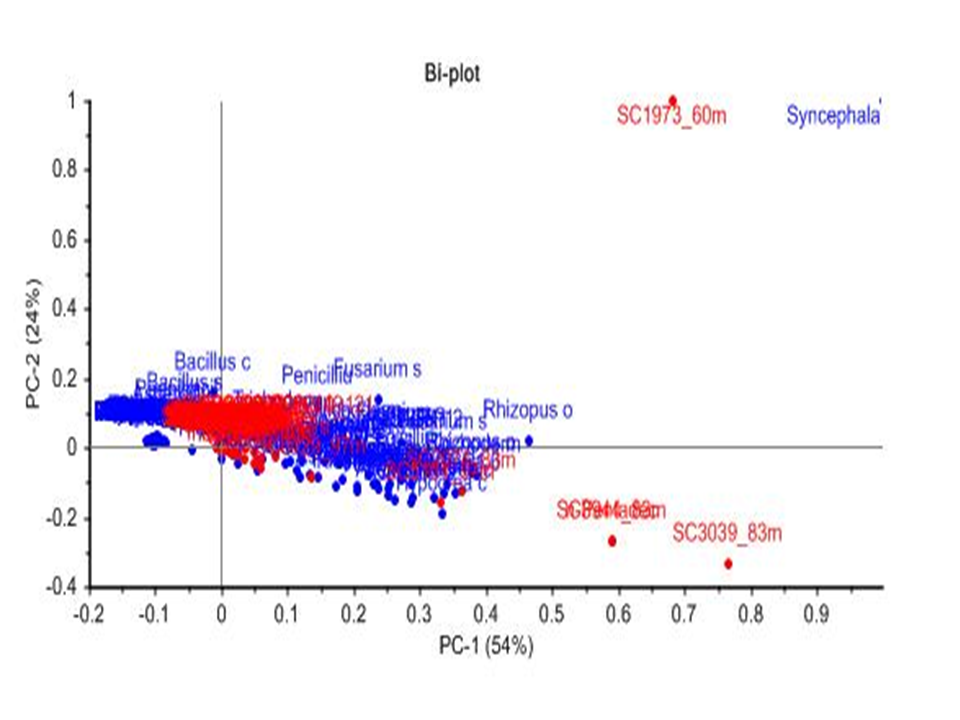

Supplement: Figure S5 — PCA plot of GC data of bacterial and fungal infected wood samples collected 3 months after artificial inoculation Bacteria Fungi. [file Image5.TIF]
