# Supplementary material for: Fungal and Bacterial Diversity Isolated from Aquilaria malaccensis Tree and Soil, Induces Agarospirol Formation within 3 Months after Artificial Infection
Source: Front Microbiol. 2017 Jul 11;8:1286. doi: 10.3389/fmicb.2017.01286 (PMC5507295; doi:10.3389/fmicb.2017.01286)
Supplement: Table S1 — List of sample collected from different districts of Assam for microbial isolation. [file Table1.PDF]

Table S1. List of sample: Samples were collected from different districts of Assam for microbial isolation.

S.No.	Location	No. of collected Samples	
		**Stem**	**Soil**
1	Amgurikhat	2	1
2	Bagmaria	3	2
3	Bhogpur	1	1
4	Chamaguri	4	4
5	Chessa	1	1
6	Dolerseria	5	6
7	Gharphalia	3	5
8	Jaipur	1	1
9	Jakaria Gaon	8	6
10	Karkhowa	1	6
11	Khanikar	3	2
12	Khatowal	2	2
13	Laluk	1	1
14	Madhupur	1	1
15	Mautgaon	1	3
16	Nachungi	2	2
17	Namti Chariali	4	2
18	Senchowa	3	1
19	Shotaichiga	2	1
20	Simalgudi	1	1
21	Sotaichinga	1	1
	Total	50	50
