# Supplementary material for: Fungal and Bacterial Diversity Isolated from Aquilaria malaccensis Tree and Soil, Induces Agarospirol Formation within 3 Months after Artificial Infection
Source: Front Microbiol. 2017 Jul 11;8:1286. doi: 10.3389/fmicb.2017.01286 (PMC5507295; doi:10.3389/fmicb.2017.01286)
Supplement: Table S2 — Identification of fungal and bacterial isolates on the basis of sequence similarity with different sequence data base. [file Table2.PDF]

Table S2 : Identification of fungal and bacterial isolates on the basis of sequence similarity with different sequence data base: (A) fungal sequences were identified on the basis of National Center of Biotechnology Information (NCBI) and UNITE database and (B) Bacterial sequences were identified on the basis of NCBI and SILVA database

(A) Fungal Identification

			NCBI				UNITE		
**Culture ID**	**Accession no of submitted Sequence**	**Base**	**Similarity**	**Accession No**	**% Identity**	**E-Value**	**Similarity**	**Accession No**	**E-value**
AQGS1	KP670189	505	*Nigrospora sp*	KX219601.1	99	0	*Nigrospora*	KP670189	0
AQGS2	KP670190	255	*Arthrinium sp*	KP670190.1	100	3.00E-129	*Arthrinium hydei*	KY356087	1.00E-128
AQGS3	KP670191	592	*Hypocreales sp*	EU164804.1	98	0	*Hypocreales*	EU164804	0
AQGS4	KP670192	480	*Mucor sp.*	GQ220709.1	91	0	*Mucor circinelloides*	KF742382	0
AQGS5	KP670193	562	*Trichoderma harzianum*	KC847182.1	99	0	*Trichoderma harzianum*	KC847182	0
AQGS6	KP670194	470	*Hypocrea caerulescens*	KF313113.1	100	0	*Trichoderma caerulescens*	KF313113	0
AQGS7	KP670195	364	*Epicoccum sorghinum*	KX611655.1	100	0	***Phoma***	KY046264	0
AQGS8	KP670196	418	*Fusarium incarnatum*	KX965656.1	100	0	*Fusarium incarnatum*	KR017874	0
AQGS9	KP670197	460	*Fusarium sp*	HQ696053.1	100	0	*Fusarium*	HQ696053	0
AQGS10	KP670198	480	*Lasiodiplodia pseudotheobromae*	KF766193.1	97	0	*Lasiodiplodia*	KM266280	0
AQGS12	KP721601	448	*Lasiodiplodia theobromae*	KY952689.1	100	0	*Lasiodiplodia theobromae*	LN849073	0
AQGS13	KP721578	1108	*Gibberella intermedia*	HQ443247.1	92	0	*Fusarium*	KP721578	0
AQGS14	KP721602	502	*Curvularia sp*	KT012655.1	100	0	*Curvularia*	KT012655	0
AQGS16	KP721603	446	*Lasiodiplodia theobromae*	KY952689.1	100	0	*Lasiodiplodia theobromae*	LN849073	0
AQGS17	KP721604	638	*Mucor circinelloides*	KX349456.1	100	0	*Mucor circinelloides*	KX349464	0
AQGS18	KP721605	490	*Fusarium equiseti*	KJ412506.1	100	0	*Fusarium*	KC354530	0
AQGS19	KP721577	455	*Aschersonia sp*	KF675752.1	100	0	*Aschersonia*	KF675752	0
AQGS 20	KR364887	517	*Aschersonia sp*	KF679355.1	100	0	*Aschersonia*	KR364887	0
AQGS21	KP721606	425	*Fusarium solani*	KU377470.1	100	0	*Fusarium solani*	KU377470	0
AQGS22	KP721576	839	*Epicoccum nigrum*	KM977751.1	97	0	*Epicoccum nigrum*	KM977751	0
AQGS23	KP721607	455	*Aschersonia sp.*	KF675752.1	100	0	*Aschersonia sp.*	KF675752	0
AQGS24	KP721608	484	*Fusarium oxysporum*	HQ647333.1	100	0	*Fusarium oxysporum*	HQ647333	0
AQGS25	KP721609	483	*Fusarium equiseti*	KR709055.1	100	0	*Gibberella intricans*	KR709055	0
AQGS26	KP721610	503	*Aporospora sp.*	KU571502.1	99	0	*Aporospora sp. (Botryosphaeriaceae)*	KU571502	0
AQGS27	KP721611	579	*Aspergillus nomius*	KR905619.1	95	0	*Aspergillus flavus*	JX514875	0
AQGS28	KP721575	681	*Trichoderma longibrachiatum*	KY568698.1	94	0	*Trichoderma longibrachiatum*	JN039069	0

AQGS29	KP721612	560	*Curvularia verruculosa*	KJ748697.1	99	0	*Curvularia verruculosa*	KJ748697	0
AQGS30	KP721613	556	*Penicillium citrinum*	KM070816.1	98	0	*Penicillium citrinum*	KM070816	0
AQGS31	KP721574	732	*Epicoccum nigrum*	KY318482.1	97	0	*Epicoccum nigrum*	KR912314	0
AQGS32	KP721573	324	*Diaporthe phaseolorum*	KR017034.1	98	3.00E-159	***Phomopsis***	KX065020	9.00E-158
AQGS33	KP721572	450	*Fusarium sp*	KU527799.2	100	0	*Gibberella indica*	KM052902	0
AQGS34	KP721617	490	*Epicoccum sp*	GU973785.1	99	0	*Epicoccum*	KC329633	0
AQGS35	KP721571	615	*Fusarium sp*	GQ352485.1	97	0	*Fusarium*	GQ352485	0
AQGS38	KP721562	783	*Penicillium chrysogenum*	KP278166.1	92	0	*Penicillium chrysogenum*	KR912332	0
AQGS39	KP721570	536	*Gibberella sp*	KP721578.1	100	0	*Gibberella fujikuroi*	KU258729	0
AQGS40	KP721569	569	*Aspergillus sp.*	KY587304.1	97	0	*Aspergillus*	KY587304	0
AQGS41	KP721568	704	*Curvularia lunata*	KX156939.1	99	0	*Curvularia lunata*	KX156939	0
AQGS42	KP721614	372	*Epicoccum nigrum*	KX138420.1	100	0	*Epicoccum nigrum*	KX138420	0
AQGS43	KP721567	467	*Epicoccum nigrum*	KT192212.1	100	0	*Epicoccum nigrum*	KT192212	0
AQGS44	KP721566	708	*Fusarium oxysporum*	KU671036.1	95	0	*Fusarium*	KU671036	0
AQGS45	KP721565	518	*Aspergillus sp.*	KU527791.2	99	0	*Aspergillus*	KX953397	0
AQGS46	KP721564	329	*Fusarium oxysporum*	KY319345.1	99	4.00E-168	*Fusarium*	LT719152	7.00E-166
AQGS47	KP721615	459	*Lasiodiplodia theobromae*	KP998517.1	100	0	*Lasiodiplodia theobromae*	KX650845	0
AQGS48	KP721563	910	*Aspergillus sp*	KR154911.1	100	0	*Aspergillus*	KR154911	0
AQGS49	KP721561	772	*Lasiodiplodia theobromae*	LC074359.1	96	0	*Lasiodiplodia theobromae*	LC074359	0
AQGS50	KP721616	385	*Diaporthe sp*	KC357558.1	98	0	*Diaporthe sp*	KC357560	0
AQGWD1	KP721625	542	*Trichoderma virens*	KY225679.1	100	0	*Trichoderma virens*	LT220743	0
AQGWD2	KP721626	495	*Penicillium chrysogenum*	KX421460.1	100	0	*Penicillium chrysogenum*	NR_077145	0
AQGWD3	KP721627	327	*Geotrichum candidum*	KT175200.1	100	2.00E-162	*Geotrichum candidum*	KT175200	2.00E-167
AQGWD4	KP721628	492	*Trichoderma atroviride*	KY225662.1	99	0	*Trichoderma*	KU504286	0
AQGWD5	KP721592	644	*Lasiodiplodia theobromae*	LC074359.1	96	0	*Lasiodiplodia theobromae*	LC074359	0
AQGWD6	KP721591	464	*Alternaria alternata*	MF029625.1	100	0	*Alternaria tenuissima*	LT799975	0
AQGWD7	KP721590	868	*Pichia sp.*	KT987926.1	96	0	*Pichia*	KT987926	0
AQGWD8	KP721589	526	*Mucor circinelloides*	JF439687.1	99	0	*Mucor circinelloides*	JF439688	0
AQGWD9	KP721629	464	*Fusarium oxysporum*	KY319356.1	100	0	*Fusarium oxysporum*	X94173	0
AQGWD10	KP721588	530	*Meyerozyma guilliermondii*	KX580709.1	99	0	*Meyerozyma guilliermondii*	LN626312	0
AQGWD11	KP721587	373	*Mucor circinelloides*	KR091781.1	100	0	*Mucor circinelloides*	KX928837	0
AQGWD12	KP721630	533	*Hypocrea rufa*	KC012451.1	100	0	*Trichoderma viride*	KC012451	0
AQGWD13	KP721586	680	*Paecilomyces sp.*	KF811433.1	86	0	*Paecilomyces*	KF811433	0
AQGWD14	KP721585	569	*Trichoderma longibrachiatum*	KX463453.1	99	0	*Trichoderma viride*	KY354581	0
AQGWD 15	KP721584	860	*Hypocrea lixii*	FR872742.1	94	0	*Trichoderma harzianum*	KC576642	0
AQGWD17	KP721583	1303	*Aspergillus sp.*	KP686456.1	94	0	*Aspergillus flavus*	KM113843	0
AQGWD18	KP721582	1124	*Penicillium griseofulvum*	MF034654.1	86	0	*Penicillium chrysogenum*	KR912333	0
AQGWD19	KP721581	762	*Fusarium sp.*	KP721580.1	98	0	*Gibberella indica*	KM052902	0
AQGWD20	KP721580	489	*Fusarium proliferatum*	KJ767073.1	99	0	*Fusarium proliferatum*	KJ767073	0

AQGWD21	KP721579	344	*Fusarium solani*	KR017036.1	100	1.00E-178	*Fusarium solani*	KR017036	0
AQGSS1	KP721618	414	*Penicillium sp.*	KX611014.1	99	0	*Penicillium citrinum*	LT558897	0
AQGSS2	KP721619	415	*Talaromyces aculeatus*	KP178536.2	100	0	*Talaromyces aculeatus*	KP178536	0
AQGSS3	KP721600	660	*Alternaria tenuissima*	KU937315.1	90	0	*Alternaria lini*	KP341702	0
AQGSS4	KP721620	361	*Aspergillus sydowii*	KX894659.1	99	0	*Aspergillus*	LC133814	0
AQGSS5	KP721621	420	*Aspergillus aculeatus*	EU645743.1	97	3.00E-06	***Talaromyces verruculosus***	KM507166	3.00E-108
AQGSS6	KP721622	511	*Aspergillus flavus*	KY437092.1	100	0	*Aspergillus flavus*	LC195001	0
AQGSS8	KP721623	525	*Trichoderma longibrachiatum*	KX463453.1	100	0	*Trichoderma viride*	KY354581	0
AQGSS9	KP721599	490	*Talaromyces aculeatus*	KP178536.2	99	0	*Talaromyces aculeatus*	KP178536	0
AQGSS10	KP721598	882	*Aspergillus sp*	KP686456.1	95	0	*Aspergillus sp*	KP686456	0
AQGSS11	KP721597	596	*Trichoderma asperellum*	KY810796.1	99	0	*Trichoderma asperellum*	KY810796	0
AQGSS12	KP721596	1047	*Syncephalastrum racemosum*	KX815982.1	100	0	*Syncephalastrum racemosum*	KX815982	0
AQGSS13	KP721624	458	*Aspergillus flavus*	KY684266.1	100	0	*Aspergillus flavus*	LN482587	0
AQGSS14	KP721595	493	*Fusarium proliferatum*	KU527804.2	100	0	*Fusarium proliferatum*	X94171	0
AQGSS15	KP721594	730	*Alternaria tenuissima*	KX664408.1	96	0	*Alternaria tenuissima*	KX664408	0
AQGSS16	KP721593	352	*Aspergillus flavus*	LT604472.1	86	1.00E-89	*Aspergillus flavus*	LT604472	8.00E-96
AQGSS17	KP721618	630	*Aspergillus flavipes*	KX674601.1	87	1.00E-150	*Aspergillus flavipes*	KX674601	1.00E-158

(B) Bacterial Identification

			**NCBI**				**SILVA**		
**Culture ID**	**Accession no of submitted Sequence**	**Base**	**Similarity**	**Accession No**	**% Identity**	**E-Value**	**Similarity**	**Accession No**	**% Identity**
AQGSSB2	KP886465	931	*Lysinibacillus sp.*	KX350196.1	99	0	*Lysinibacillus varians*	CP006837	99.68
AQGSSB5	KP886466	804	*Bacillus megaterium*	LC259118.1	100	0	*Bacillus megaterium WSH-002*	CP003017	100
AQGSSB6	KP886467	947	*Bacillus meqaterium*	KY316436.1	99	0	*Bacillus megaterium WSH-002*	CP003017	99.58
AQGSSB8	KP886468	879	*Bacillus aryabhattai*	KY316449.1	100	0	*Bacillus megaterium*	GQ284474	100
AQGSSB12	KP886469	990	*Pseudomonas aeruginosa*	CP015650.1	99	0	*Pseudomonas aeruginosa BL23*	AXPD01000031	99.8
AQGSSB13	KP886470	764	*Bacillus thuringiensis*	KY203801.1	99	0	*Bacillus thuringiensis serovar*	CP004123	99.35

AQGSSB14	KP886471	677	*Bacillus sp*	AY663659.1	99	0	*Bacillus megaterium WSH-002*	CP003017	98.97
AQGSSB15	KP886472	792	*Paenibacillus sp*	KU041667.1	100	0	*Paenibacillus sp. YXA3-5*	JF701948	99.12
AQGSSB19	KP886473	774	*Bacillus pumilus*	KY818924.1	99	0	*Bacillus pumilus*	CP011150	99.23
AQGSSB20	KP886474	724	*Lysinibacillus xylanilyticus*	KY316401.1	99	0	***Bacillus sp.***	HM233997	98.9
AQGWDB1	KP886475	917	*Pantoea dispersa*	KT149750.1	100	0	*Pantoea dispersa EGD-AAK13*	AVSS01000032	98.69
AQGWDB4	KP886476	792	*Bacillus thuringiensis*	CP021061.1	99	0	*Bacillus thuringiensis serovar*	CP004123	99.87
AQGWDB5	KP886477	884	*Bacillus meqaterium*	KY316436.1	99	0	*Bacillus sp. MB50*	AB518959	99.77
AQGWDB6	KP886478	836	*Bacillus subtilis*	KY511695.1	100	0	*Bacillus subtilis BSn5*	CP002468	99.66
AQGWDB9	KP886479	636	*Bacillus sp*	MF004206.1	100	0	*Bacillus cereus*	CP008712	100
AQGWDB10	KP886480	700	*Bacillus vietnamensis*	KY321373.1	100	0	*Bacillus sp. AK1882*	FJ573190	99.86
AQGWDB12	KP886481	969	*Stenotrophomonas*	KF479689.1	100	0	*Stenotrophomonas maltophilia*	GU130529	99.79
AQGWDB13	KP886482	753	*Bacillus cereus*	CP020937.1	99	0	*Bacillus cereus*	AB680417	99.6
AQGWDB14	KP886483	878	*Bacillus subtilis*	MF040290.1	100	0	*Bacillus subtilis BSn5*	CP002468	100
AQGWDB15	KP886484	694	*Bacillus thuringiensis*	KT823940.1	99	0	*Bacillus thuringiensis serovar*	AE017355	99.71
AQGWDB16	KP886485	811	*Pseudomonas aeruginosa*	KP866921.1	100	0	*Pseudomonas aeruginosa*	AB830081	99.88
AQGWDB18	KP886486	894	*Pseudomonas aeruginosa*	KX756232.1	100	0	*Pseudomonas aeruginosa BWHPSA048*	AZZF01000008	99.89
AQGWDB20	KP886487	327	*Bacillus thuringiensis*	KY203801.1	100	3.00E-169	*Bacillus thuringiensis*	CP013274	100
AQGSB1	KR364888	640	*Bacillus cereus*	AJ853737.1	95	0	*bacterium K2-46*	AY345416	94.62
AQGSB2	KR364889	726	*Roseomonas aerophila*	NR_109678.1	100	0	*Roseomonas musae*	AB594201	97.25
AQGSB3	KR364890	640	*Bacillus aerophilus*	KT719742.1	98	0	*Bacillus sp. NBRC 101222*	AB681408	98.13
AQGSB5	KR364891	792	*Bacillus sp.*	KU597577.1	100	0	*Bacillus thuringiensis serovar*	CP004123	98.61
AQGSB6	KR364892	524	*Bacillus subtilis*	EU124568.1	99	0	*Bacillus subtilis subsp.*	AP011541	97.11
AQGSB7	KR364893	787	*Bacillus sp*	KR185899.1	99	0	*Bacillus pseudomycoides*	AB738782	98.73
AQGSB9	KR364894	791	*Bacillus cereus*	KX035069.1	100	0	*Bacillus thuringiensis serovar*	CP004123	98.61
