# Supplementary material for: Fungal and Bacterial Diversity Isolated from Aquilaria malaccensis Tree and Soil, Induces Agarospirol Formation within 3 Months after Artificial Infection
Source: Front Microbiol. 2017 Jul 11;8:1286. doi: 10.3389/fmicb.2017.01286 (PMC5507295; doi:10.3389/fmicb.2017.01286)
Supplement: Table S3 — Per cent composition of compounds in oleoresin extract identified by gas chromatography mass spectroscopy. [file Table3.PDF]

Table S3: Percent composition of compounds in oleoresin extract identified by (GC-MS) gas chromatography mass spectroscopy

**Strains**	**% extract (w/w)**	**Values in %**				
		**2,3 Butane Diol**	**Benzyl acetone**	**Agarospirol**	**Anisyl acetone (AA)**	**Infection Length (Cm)**
Aspergillus flavus AQGSS 10	1.91	0.17	0.25	0.00	0.33	11.00
Aspergillus flavipes AQGSS 17	1.09	0.00	0.13	0.00	0.33	2.80
Aspergillus flavus AQGSS 6	0.44	0.00	0.00	0.00	0.36	3.50
Alternaria lini AQGGR1.5	2.18	0.00	0.10	0.00	1.11	2.60
Aspergillus oryzae AQGWD 17	1.65	1.63	0.00	0.00	0.33	2.00
AQGGR1.6	3.19	0.17	0.16	0.00	0.89	3.60
AQGSS 5	1.24	0.00	0.06	0.00	0.30	3.40
AQGSSB10	1.43	0.08	0.00	0.00	0.55	5.78
AQGSSB17	1.19	0.00	0.00	0.00	0.21	3.50
AQGSSB22	1.56	0.00	0.00	0.00	0.27	4.15
AQGSSB23	1.13	0.00	0.11	0.06	1.25	2.00
AQGWDB21	0.00	0.00	0.00	0.00	0.07	2.88
AQGWDB7	0.00	0.24	0.10	0.05	0.27	3.33
Aspergillus sydowii AQGSS 4	1.70	1.21	0.00	0.00	0.00	3.60
Aspergillus flavus AQGSS 6	1.66	0.00	0.00	0.00	0.17	8.00
Alternaria sp. AQGSS 3	1.97	0.00	0.11	0.00	0.39	2.00
Bacillus anthricus AQGSSB4	1.96	0.75	0.10	0.00	0.84	3.00
Bacillus anthricus strain AQGWDB2	0.00	0.23	0.04	0.05	0.26	2.91

Bacillus cereus AQGWDB13	0.61	0.00	0.02	0.00	0.83	2.53
Bacillus cereus AQGWDB9	0.00	0.20	0.21	0.09	0.46	3.45
Bacillus megaterium AQGSSB 5	1.64	0.44	0.09	0.00	0.72	3.31
Bacillus megaterium AQGWDB 3	0.00	0.98	0.05	0.03	0.23	2.35
Bacillus sp. AQGWDB10	1.61	2.08	0.05	0.00	1.15	3.10
Bacillus sp. AQGWDB4	0.00	0.12	0.18	0.00	0.71	1.86
Control	1.10	0.00	0.00	0.00	0.27	1.45
Fusarium proliferatum AQGWD 20	0.93	0.00	0.00	0.00	0.33	2.00
Fusarium solani AQGGR1.7	2.63	2.95	0.23	0.00	10.06	5.00
Lysinibacillus sp. AQGSSB20	1.17	0.32	0.00	0.00	0.34	3.60
Penicillium aethiopicum AQGGR1.2	3.05	0.17	0.15	0.67	1.07	3.00
Penicillium citrinum AQGSS 1	2.66	0.00	0.75	0.00	0.50	2.00
Pantoea dispersa AQGWDB1	1.17	1.06	0.21	3.77	0.92	1.26
Pichia kudriavzevii AQGWD 7	1.00	0.00	0.00	0.00	0.12	3.00
Penicillium polonicum AQGGR1.1	5.39	0.00	1.25	3.33	0.00	2.60
Pseudomonas aeruginosa AQGWDB16	0.00	0.02	0.06	0.00	0.43	4.55
Paenibacillus alvei voucher AQGSSB15	1.24	0.02	0.03	0.00	0.49	11.83
Rhizopus oryzae AQGGR1.3	2.19	0.00	0.18	0.05	0.95	2.00
Syringe control	0.91	0.00	0.00	0.00	0.17	1.80
Syncephalastrum racemosum AQGSS 12	1.89	0.00	1.00	1.34	1.17	2.60
Syringe control	1.28	0.00	0.00	0.00	0.37	1.80

Trichoderma asperellum AQGSS 11	1.15	0.00	0.00	0.20	0.30	3.00
Talaromyces aculeatus AQGSS 2	1.97	0.00	0.00	0.00	0.13	2.10
Trichoderma harzianum AQGGR1.4	2.41	0.00	0.10	0.00	1.15	2.00
Trichoderma koningii AQGWD 4	1.74	1.84	0.00	0.00	0.17	2.00
wood control	0.29	0.00	0.00	0.00	0.00	0.00
